# Evaluation of a novel central venous access port for direct catheter insertion without a peel-away sheath

**DOI:** 10.1007/s11604-024-01658-5

**Published:** 2024-09-17

**Authors:** Toshihiro Iguchi, Takahiro Kawabata, Yusuke Matsui, Koji Tomita, Mayu Uka, Noriyuki Umakoshi, Soichiro Okamoto, Kazuaki Munetomo, Takao Hiraki

**Affiliations:** 1 Department of Radiology, Okayama University Hospital, 2-5-1 Shikata-Cho Kita-Ku, Okayama, 700-8558 Japan; 2 Department of Radiological Technology, Faculty of Health Sciences, Okayama University, 2-5-1 Shikata-Cho Kita-Ku, Okayama, 700-8558 Japan; 3 Department of Radiology, Faculty of Medicine, Dentistry and Pharmaceutical Sciences, Okayama University, 2-5-1 Shikata-Cho Kita-Ku, Okayama, 700-8558 Japan

**Keywords:** Central venous catheters, Vascular access device, Treatment outcome, Safety

## Abstract

**Purpose:**

This study retrospectively evaluated the feasibility and safety of implanting a newly developed central venous access port (CV-port) that allows catheter insertion into a vein without the use of a peel-away sheath, with a focus on its potential to minimize risks associated with conventional implantation methods.

**Materials and methods:**

All procedures were performed using a new device (P-U CelSite Port™ MS; Toray Medical, Tokyo, Japan) under ultrasound guidance. The primary endpoint was the implantation success rate. The secondary endpoints were the safety and risk factors for infection in the early postprocedural period (< 30 days).

**Results:**

We assessed 523 CV-port implantations performed in a cumulative total of 523 patients (240 men and 283 women; mean age, 61.6 ± 13.1 years; range, 18–85 years). All implantations were successfully performed using an inner guide tube and over-the-wire technique through 522 internal jugular veins and one subclavian vein. The mean procedural time was 33.2 ± 10.9 min (range 15–112 min). Air embolism, rupture/perforation of the superior vena cava, or hemothorax did not occur during catheter insertion. Eleven (2.1%) intraprocedural complications occurred, including Grade I arrhythmia (n = 8) and subcutaneous bleeding (n = 1), Grade II arrhythmia (n = 1), and Grade IIIa pneumothorax (n = 1). Furthermore, 496 patients were followed up for ≥ 30 days. Six early postprocedural complications were encountered (1.1%), including Grade IIIa infection (n = 4), catheter occlusion (n = 1), and skin necrosis due to subcutaneous leakage of trabectedin (n = 1). These six CV-ports were withdrawn, and no significant risk factors for infection in the early postprocedural period were identified.

**Conclusion:**

The implantation of this CV-port device demonstrated comparable success and complication rates to conventional devices, with the added potential benefit of eliminating complications associated with the use of a peel-away sheath.

## Introduction

Recently, the number of patients undergoing central venous access port (CV-port) implantation for various indications, including systemic chemotherapy and total nutrition, has increased remarkably [1]. Simultaneously, technological advances have yielded superior and improved devices, such as catheters, that are less prone to breakage and ports with improved pressure resistance. The implantation procedure has also changed from the surgical cutdown method and blinded venous puncture to image-guided venous puncture (real-time ultrasound and fluoroscopic guidance) for less invasive, safer, and more accurate implantation.

Traditionally, in the CV-port implantation procedure, a guidewire is advanced following target vessel puncture, and subsequently, a peel-away sheath is inserted along the guidewire [2, 3]. Furthermore, the catheter is advanced through this sheath into the superior vena cava (SVC), and the sheath is peeled away. However, this method (catheter insertion via peel-away sheath) raises several concerns, viz. the creation of a hole larger than the catheter at the venous entry site, a gap between the catheter and sheath, and a temporary direct connection between the vascular lumen with the external environment. Studies have reported complications associated with the use of peel-away sheaths, including air embolism, SVC rupture, and hemothorax [4, 5], as well as the potential risk of increased bleeding from the venous puncture site.

A new CV-port device (P-U CelSite Port™ MS; Toray Medical, Tokyo, Japan) became commercially available in Japan in 2019. With this device, a catheter is advanced along the guidewire (the over-the-wire technique) without using a peel-away sheath. Approximately 7% of patients who undergo image-guided central venous access experience complications (major and minor) [6]. We hypothesized that if the success and complication rates of this new CV-port device were comparable to those of conventional devices, it could offer additional benefits, such as eliminating the risk of complications associated with the use of a peel-away sheath. Empirically, in the clinical setting, this CV-port is comparable with conventional devices in terms of feasibility of implantation and safety. However, detailed evaluations of its clinical outcomes remain limited. Therefore, this study aims to retrospectively evaluate the feasibility and safety of implanting this novel CV-port device, with a focus on its potential to minimize risks associated with conventional implantation methods.

## Materials and methods

This retrospective study was conducted in accordance with the Declaration of Helsinki and approved by our institution’s ethics committee (approval number: KEN2405-007). Before the procedure, written informed consent for CV-port implantation was obtained from all patients. Opt-out consent was obtained for the retrospective use of the patients’ data.

### Patients

Our institution’s medical records were reviewed retrospectively. This study’s inclusion criteria were as follows: i) patients who underwent CV-port implantation between May 2019 and May 2023 at our department, ii) implantation was attempted through the internal jugular vein (IJV), and iii) age ≥ 18 years. The exclusion criteria were as follows: i) implantation with a CV-port other than the P-U CelSite Port™ MS or ii) patients undergoing withdrawal and implantation of a CV-port simultaneously in one session.

### Study endpoints

The primary endpoint was the implantation success rate. The secondary endpoints were the safety and risk factors for the occurrence of infection in the early postprocedural period (< 30 days). Primary success was defined as successful implantation through a punctured IJV. Secondary success was defined as successful implantation through another vein (viz. the contralateral IJV, subclavian vein, brachial vein, or others) and not the initially punctured IJV. Safety was assessed by evaluating complications using the Clavien-Dindo classification [7]. Complications were divided into intraprocedural and early postprocedural complications. We focused on the occurrence of air embolism, SVC rupture/perforation, and hemothorax during catheter insertion. Air embolization was assessed by audible air aspiration during catheter insertion or by fluoroscopic visualization of air within the right atrium or pulmonary artery [4]. The following factors were evaluated as potential risk factors for the occurrence of infection in the early postprocedural period: patient characteristics (age, sex, body mass index, and hospitalization status), preimplantation laboratory investigations (white blood cells, platelets, C-reactive protein, and prothrombin time-international normalized ratio), and procedure-related factors (punctured IJV, operator experience, and procedural time). Procedural time was defined as the period between the administration of local anesthesia and the last chest radiograph.

### P-U CelSite Port™ MS

**Fig. 1:**
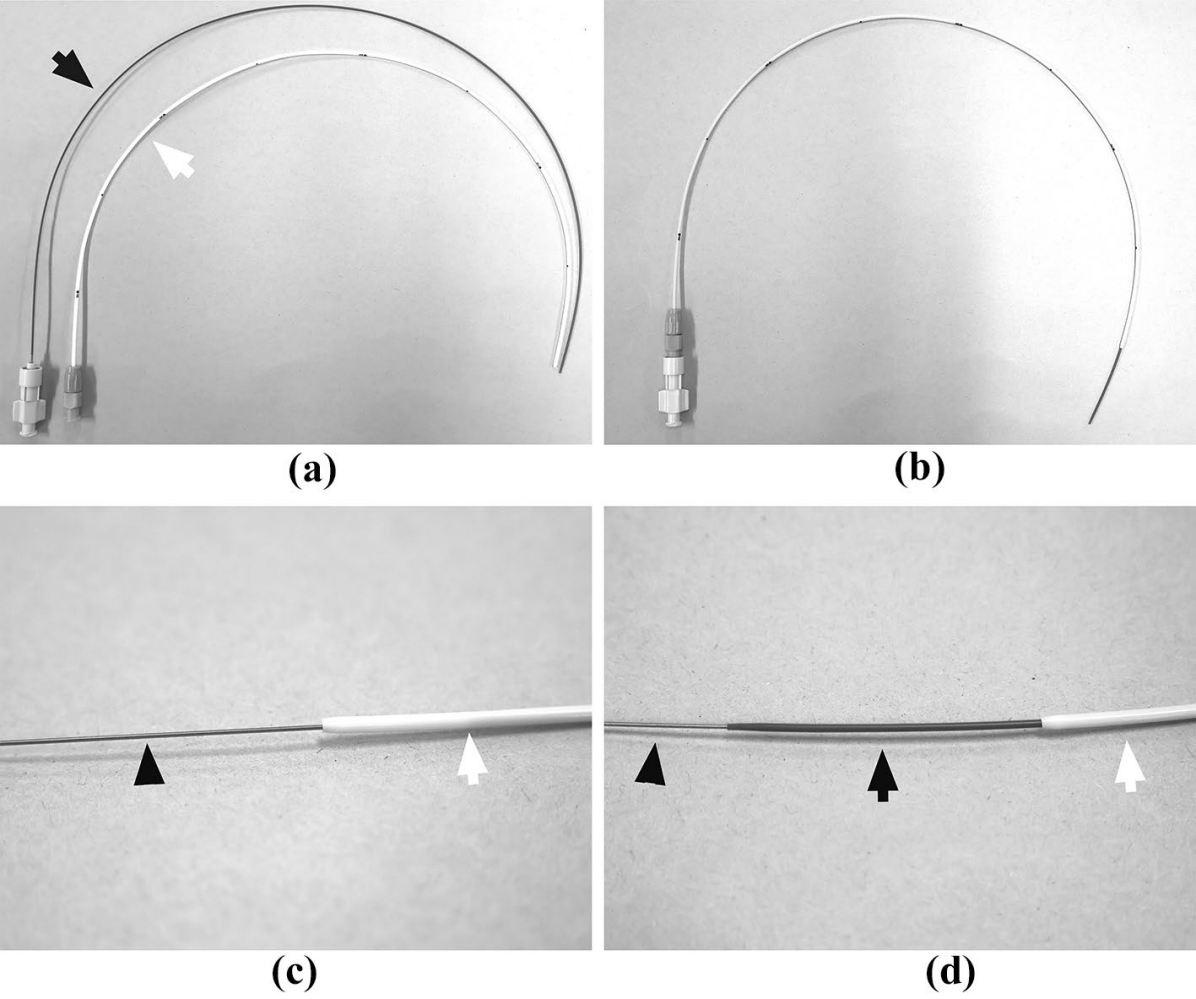
P-U CelSite Port™ MS. **a** Photo shows an inner guide tube (black arrow) and a 5-F 40-cm catheter (white arrow). **b** The inner guide tube is inserted into the catheter to reduce the gap between the guidewire and catheter and advanced into the punctured vein. **c** Without an inner guide tube, a large gap is created between the catheter (white arrow) and guidewire (arrowhead). **d** The tip of the inner guide tube is tapered to reduce insertion resistance and improve catheter straightness. By inserting the inner guide tube (black arrow) into the catheter (white arrow), the gaps between the guidewire (arrowhead) and inner guide tube, as well as between the inner guide tube and catheter, are eliminated

This device features a special inner guide tube combined with a catheter (Anthrone® P-U catheter; Toray Medical), which enables catheter insertion without the use of a peel-away sheath (Fig. 1). Inserting an inner guide tube reduces the gap between the guidewire and the catheter. The tips of both the inner guide tube and catheter are tapered to minimize insertion resistance and improve catheter straightness. The size of the hole at the venous puncture site is reduced by approximately 50% compared to the conventional method (i.e., using a peel-away sheath) [8].

### CV-port implantation procedure

A board-certified interventional radiologist (an expert) or a radiology trainee under the direct supervision of an expert in an interventional radiology suite performed the implantations under conscious sedation. The right IJV was typically the first choice. Anticoagulants and antiplatelet therapy were generally not discontinued. Oral prophylactic antibiotics were usually started on the day of the procedure and continued for 3 days.

**Fig. 2:**
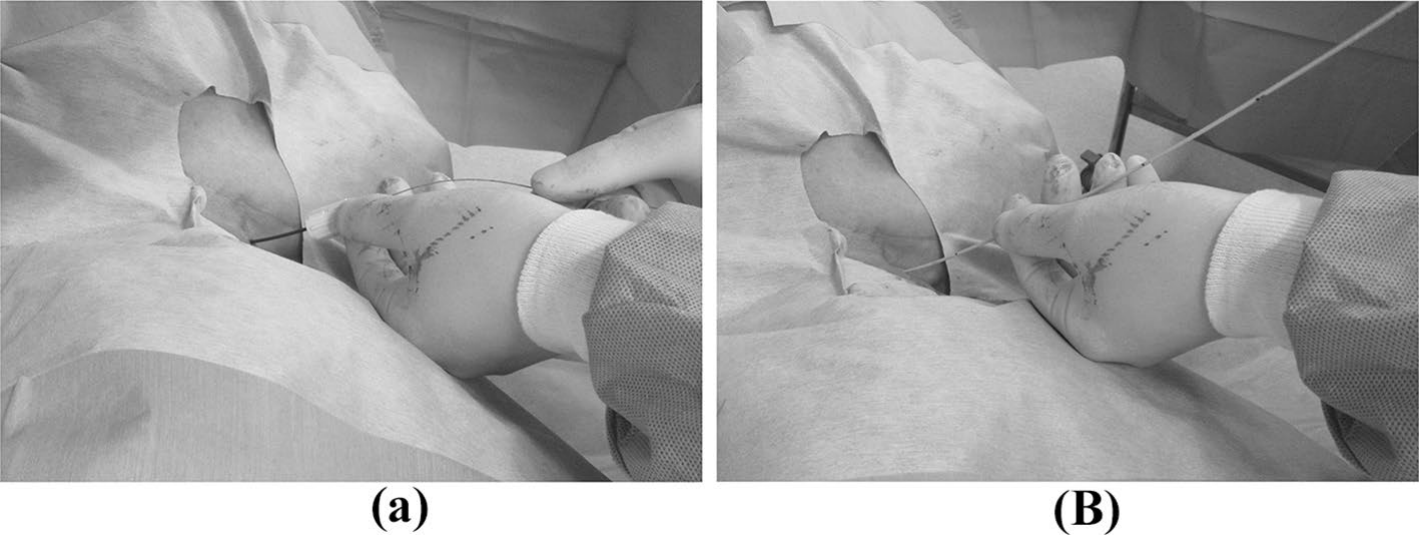
Following dilation with a 5-F dilator (**a)**, the catheter is inserted into the punctured internal jugular vein using over-the-wire technique (**b)**

First, an ultrasound evaluation of the IJV was performed before skin antisepsis to screen the patency and size of the vein and rule out venous thrombosis. After local anesthesia with 1% lidocaine, the selected IJV was punctured using a short 22-gauge cannula puncture needle (42 mm long) under real-time ultrasound guidance, and a 0.018-inch guidewire (80 cm long) was introduced into the inferior vena cava using fluoroscopy. Following dilation with a 5-F dilator, a 5-F catheter with an inner tube was advanced under fluoroscopic guidance until its tip reached the SVC along the guidewire (over-the-wire technique) without the use of a peel-away sheath (Fig. 2). The inner guide tube and guidewire were then removed. Furthermore, a subcutaneous pocket was created to implant the port beneath the subclavian area. The catheter was then passed from the venous puncture site to the pocket through the subcutaneous tissue using a metal tunneler and connected to the port (Celcite®; B. Braun Medical, Melsungen, Germany). The patency of the port system was determined by aspirating blood and flushing it with heparinized saline without any problem. The port was sutured to the fascia, and the pocket was closed. Finally, a chest radiograph was obtained to observe the position of the catheter tip and the presence of complications, such as pneumothorax and hemothorax. According to the guideline [2], the catheter tip was located between the tracheal bifurcation site and the cavoatrial junction.

### Statistical analysis

Patients were divided into two groups based on the occurrence of infection in the early postprocedural period. The factors were compared between the two groups using Fisher’s exact test for categorical values and the Mann–Whitney U test for numerical values. SPSS version 26 (IBM Corp., Armonk, NY, USA) was used, and statistical significance was set at P < 0.05.

## Results

**Fig. 3:**
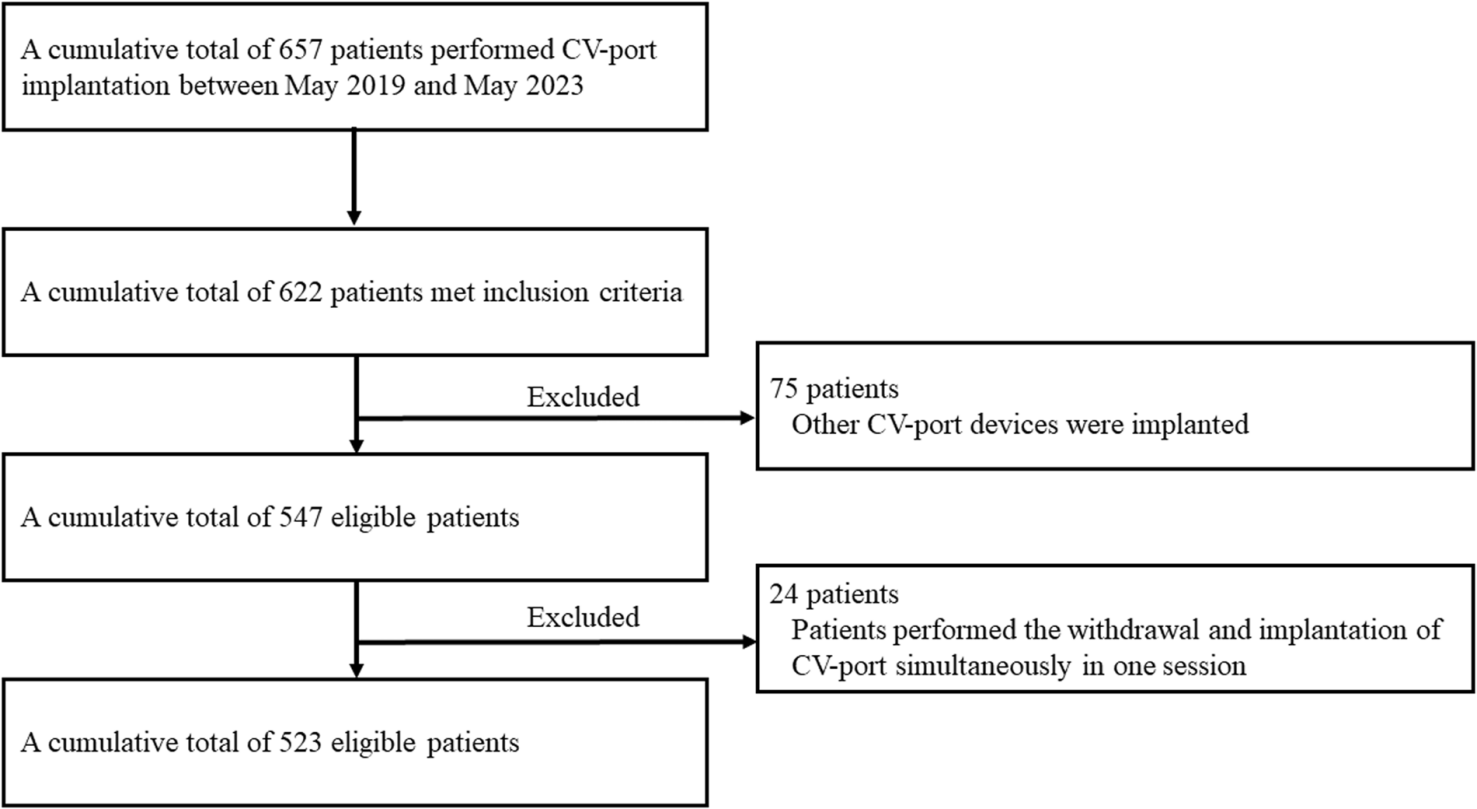
Flow chart diagram with the number of patients

Overall, 657 CV-port implantations were performed in our department between May 2019 and May 2023. Of them, 523 CV-port implantations performed in a cumulative total of 523 patients (240 men and 283 women; mean age 61.6 ± 13.1 years; range 18–85 years) met this study’s inclusion criteria (Fig. 3). All but four patients underwent implantation for systemic chemotherapy. The patient and procedure characteristics are summarized in Table 1.

**Table 1:** Characteristics of 523 patients and 523 implantations

Variable		Value
Patient		
Age (y)	Mean ± SD	61.6 ± 13.1
Gender	Man/Woman	240/283
Body mass index	Mean ± SD	22.4 ± 4.6
Hospitalization status	Inpatient/Outpatient	413/110
Preimplantation laboratory investigation		
White blood cell (× 10^3^/μL)	Mean ± SD	5.84 ± 2.86
Platelet (× 10^4^/μL)	Mean ± SD	27.3 ± 12.5
C-reactive protein (mg/dL)	Mean ± SD	1.51 ± 2.92
PT-INR*	Mean ± SD	1.03 ± 0.15
Procedure		
Punctured IJV	Right/Left	445/78
Operator experience	Trainee/Expert	349/174
Procedural time (min)	Mean ± SD	33.2 ± 10.9
Intraprocedual complication	Yes/No	11/512
Early postprocedual complication	Yes/No	6/517

*SD* standard deviation, *PT-INR* prothrombin time-international normalized ratio, *IJV* internal jugular vein

^*^PT-INR values are missing for 64 implantations

All CV-port implantations were primary successes (n = 522 [99.8%]), except for one instance of secondary success (n = 1 [0.2%]). Implantations were performed by 349 trainees and 174 experts, and the right and left IJVs were punctured first in 445 and 78 cases, respectively. The mean procedural time was 33.2 ± 10.9 min (range, 15–112 min). All but two implantations were performed under local anesthesia; two implantations were performed under general anesthesia due to ardent requests by the patients. During the longest implantation, the trainee punctured the patient’s right IJV without administering anticoagulants and successfully advanced the catheter. Subsequently, when a subcutaneous tunnel was created, a small subcutaneous artery located in the tunnel's path was injured. Even after passing the catheter from the venous puncture site to the pocket through the subcutaneous tunnel, bleeding continued from the subcutaneous tunnel. In this scenario, the catheter was in the way, and manual compression failed to stop the bleeding; therefore, the catheter was withdrawn completely, and manual compression of the subcutaneous tunnel route successfully stopped the bleeding (Grade 1 complication). The right subclavian vein was then punctured, and the CV-port was successfully implanted without any problems (secondary success).

No cases of air embolism, SVC rupture/perforation, or hemothorax were encountered during catheter insertion. Eleven (2.1%) intraprocedural complications occurred, including Grade I arrhythmia (n = 8) and subcutaneous bleeding (n = 1), Grade II arrhythmia (n = 1), and Grade IIIa pneumothorax (n = 1) (Table 2). Follow-up for ≥ 30 days was completed in 496 patients. Six early postprocedural complications occurred (1.1%), including Grade IIIa infection (n = 4), catheter occlusion (n = 1), and skin necrosis due to subcutaneous leakage of trabectedin (n = 1) (Table 2). These six CV-ports were withdrawn, and no significant risk factors for infection in the early postprocedural period were identified (Table 3).

**Table 2:** Summary of complications

Grade	Complication	Number
Intraprocedural		11 (2.1)
Grade I	Arrhythmia	8 (1.5)
	Subcutaneous bleeding	1 (0.2)
Grade II	Arrhythmia	1 (0.2)
Grade IIIa	Pneumothorax	1 (0.2)
Early postprocedural (< 30 days)		6 (1.1)
Grade IIIa	Infection	4 (0.8)
	Catheter occlusion	1 (0.2)
	Skin necrosis	1 (0.2)

Numbers in parentheses are percentages

**Table 3:** Variables in infection and non-infection groups in 523 implantations

Variable		Infection (n = 4)	Non-infection (n = 519)	P-value
Patient				
Age (y)	Mean ± SD	69.8 ± 3.4	61.5 ± 13.1	0.225
Gender	Man/Woman	1/3	239/280	0.629
Body mass index	Mean ± SD	21.3 ± 3.3	22.4 ± 4.6	0.643
Hospitalization status	Inpatient/Outpatient	2/2	411/108	0.196
Preimplantation laboratory investigation				
White blood cell (× 10^3^/μL)	Mean ± SD	4.93 ± 3.06	5.84 ± 2.86	0.379
Platelet (× 10^4^/μL)	Mean ± SD	20.7 ± 7.0	27.3 ± 12.5	0.213
C-reactive protein (mg/dL)	Mean ± SD	1.49 ± 1.49	1.51 ± 2.93	0.391
PT-INR*	Mean ± SD	0.95 ± 0.04	1.03 ± 0.15	0.062
Procedure				
Punctured IJV	Right/Left	3/1	442/77	0.477
Operator experience	Trainee/Expert	4/0	345/174	0.307
Procedural time (min)	Mean ± SD	37.8 ± 11.6	33.2 ± 10.9	0.337

*SD* standard deviation, *PT-INR* prothrombin time-international normalized ratio, *IJV* internal jugular vein

^*^PT-INR values are missing for 64 implantations in the non-infection group

## Discussion

This new CV-port system, which uses an inner guide tube and over-the-wire technique to advance the catheter into the SVC, was successfully used in all implantations with a success rate similar to that of conventional systems using a peel-away sheath. Except for one procedure involving the pneumothorax and tube placement, no major intraprocedural complications and symptoms of bleeding from the puncture site or complications associated with the use of peel-away sheaths (air embolism, SVC rupture/perforation, and hemothorax) were encountered. This CV-port differs from the conventional port only in the implantation method. The catheter and port are the same as the conventional one, allowing for medication administration, blood sampling, and high-pressure contrast injections for cross-sectional imaging.

Several complications, including air embolism, SVC rupture, and hemothorax, have been reportedly associated with the use of peel-away sheaths [4, 5]. Air embolism refers to air migration in the venous system through the catheter or sheath, which occurs in 0.3–1.4% of cases when a peel-away sheath is used [4, 9]. According to the Society of Interventional Radiology (SIR) guidelines, the incidence of perforation and hemothorax is 0.5–1% and 1–3%, respectively [6]. Although the guidelines fail to specify the exact number of perforations and hemothorax caused by the use of peel-away sheaths, it is likely that some instances could be attributed to the use of peel-away sheaths. In contrast, our results showed that these complications occurred in 0% of cases.

The reported success rate of image-guided (usually under real-time ultrasound guidance) implantation of a CV-port through the IJV is approximately 100% [1, 10–13], which is consistent with our new implantation method. The difficulty of this procedure was not high, and trainees performed 66.7% of the implantations. The SIR guidelines state that ultrasound-guided access confers a higher initial venous cannulation success [6]. In this study, all venous punctures were successfully performed under ultrasound guidance.

CV-port implantation is a safe procedure, and the use of real-time ultrasound guidance is recommended because it may reduce the risk of complications [14, 15]. Intraprocedural complications include bleeding, hematoma, catheter malposition, arrhythmias, pneumothorax, air embolism, perforation, hemothorax, and others [10, 11, 16, 17]. The SIR guidelines state that the suggested thresholds for major complications of image-guided central venous access in the internal jugular and subclavian vein approach are pneumothorax (4%), hematoma (4%), procedure-induced sepsis (4%), air embolism (2%), perforation (2%), and hemothorax (2%) [6]. The 11 intraprocedural complications observed were not specific to this implantation method but were common and minor, except for pneumothorax (n = 1). The incidence rate of pneumothorax was 0.2–0.5% in cases of IJV puncture [18], similar to the results of our study. We followed the SIR guidelines, which recommend that anticoagulants and antiplatelet medications should not be interrupted because this procedure targets the vein as a low-bleeding risk procedure [19]. However, intraoperative bleeding is usually not a clinical problem and does not require special treatment. The method of catheter advancement into the vein has changed in this device; however, the catheter, technique of puncturing the IJV, and guiding image used are the same as those used in the conventional method. Therefore, our CV-port patients appear to be comparable with the conventional ones regarding the frequency of late postprocedural complications (≥ 30 days).

Although central vein puncture-related deaths have also been reported, they are rare. The cause of such deaths included asphyxia due to neck hematoma associated with IJV puncture, superior mediastinal and right thoracic hemorrhage due to vertebral artery injury, circulatory changes due to pneumothorax, and fatal arrhythmia secondary to cardiac tamponade due to catheter perforation into the right ventricle [20, 21]. Thus, reducing the risk of hematoma formation at the IJV puncture site makes CV-port implantation safer. Patients with bleeding or coagulation abnormalities, as well as those on anticoagulant and/or antiplatelet medications, may particularly benefit from reduced bleeding at the venous puncture site.

Air migration in small amounts may be asymptomatic; however, migration of a large amount of air leads to cyanosis, increased respiratory rate, hypotension, and cardiac murmurs [2], culminating in neurological or fatal complications [22, 23]. Furthermore, hyperbaric oxygen therapy is indicated when the signs are severe [24]. However, there is a risk of neurological and cardiac complications in some patients (those with pulmonary arteriovenous malformations and septal defects of the heart) [22]. To reduce air embolization during CV-port implantation, operators must strive to minimize intravenous and atmospheric traffic by shortening the time between the removal of the inner peel-away sheath and the insertion of the catheter. In a study that investigated air embolism in 15 central venous catheter insertions, a peel-away sheath was used in all the procedures [4]. An in vitro study showed that aerostasis (air inflow) can occur under stressed conditions (with a wire in the sheath), even when using a valved peel-away sheath [25]. In the present implantation method (disuse of a peel-away sheath), the risk of air migration is relatively low.

This study has some limitations. First, the study was conducted retrospectively at a single center. Second, we did not compare our results with the results of CV-port implantation using a peel-away sheath. However, because all implantations were successfully performed with no major complications except for one case of pneumothorax, there seems to be no difference. Finally, this CV-port device could theoretically reduce the risk of some complications (air embolization, SVC rupture/perforation, hemothorax, and bleeding from the puncture site). However, as these complications are clinically rare, it may be difficult to statistically prove this benefit.

In conclusion, this new CV-port device demonstrated success and complication rates comparable to those of conventional devices. Therefore, it may be a desirable and safer alternative for patients requiring central venous access, offering additional potential benefits, such as the elimination of complications associated with the use of a peel-away sheath.

## Abbreviations

CV-port: Central venous access port
SVC: Superior vena cava
IJV: Internal jugular vein
SIR: Society of Interventional Radiology
